# Changes in Depressive and Anxiety Symptoms Following Participation in Mind–Body Activities in Older Adult Psychiatric Inpatients

**DOI:** 10.1192/bjo.2026.11065

**Published:** 2026-06-30

**Authors:** Alihassan Janmohamed, Alireza Janmohamed, Nanki Lamba

**Affiliations:** 1 Parklands Hospital, Basingstoke, United Kingdom; 2 King’s College London, London, United Kingdom

## Abstract

**Aims:**

Depression and anxiety in geriatric inpatients are linked to functional decline and prolonged hospital stays. While antidepressants are the standard of care, polypharmacy and poor tolerability often limit their effectiveness. This study examined whether a six-week structured mind-body programme achieved greater symptom reduction than treatment as usual (TAU) in an older adult psychiatric inpatient setting.

**Methods:**

This longitudinal, parallel, controlled study involved 26 in patients (aged 60 years or older) with a diagnosis of depression and comorbid anxiety. All participants were receiving standard antidepressant pharmacotherapy. Thirteen patients elected to participate in a structured mind-body programme, while 13 continued with TAU alone. The intervention consisted of weekly supervised sessions (3–5 sessions total based on clinical availability), integrating physical movement with mental engagement through yoga, tai chi, strength training, walking, and gardening. Symptoms were assessed via the Patient Health Questionnaire-9 (PHQ-9) and Generalised Anxiety Disorder-7 (GAD-7) at baseline and six weeks.

Data analysis involved paired-sample t-tests and Wilcoxon signed-rank tests for within-group changes, with between-group comparisons conducted using independent-sample t-tests or Mann–Whitney U tests as appropriate for the data distribution.

**Results:**

Baseline scores were statistically comparable between groups (p >0.05). Both cohorts demonstrated significant within-group reductions in symptoms over the six-week period (p <0.001). In the activity group, mean PHQ-9 scores decreased from 17.15 to 8.46(reduction 8.69, SD 3.54). In the TAU group, scores fell from 14.69 to 10.38 (reduction 4.31, SD 2.10).

Between-group analysis demonstrated a significantly greater reduction in depressive symptoms for the mind-body group (mean difference 4.38; 95% CI 1.99 to 6.77; p=0.001), with a large effect size (Hedges’ g=1.46). While anxiety symptoms improved in both groups (mean GAD-7 reduction 3.62 vs. 3.23), no significant between-group difference was observed (p=0.875; Hedges’ g=0.16).

**Conclusion:**

Participation in structured mind-body activities was associated with greater reductions in depressive symptoms among older adult psychiatric inpatients compared with treatment as usual alone. While anxiety symptoms improved in both groups, no additional benefit was observed for anxiety reduction. Although self-selection should be considered, these findings suggest mind-body programmes may represent a feasible and potentially effective non-pharmacological adjunct for late-life depression in inpatient psychiatric settings.

